# Candidates for Grade of Assistant Surgeon United States Marine Hospital Service

**Published:** 1898-09

**Authors:** 


### CANDIDATES FOR GRADE OF ASSISTANT SURGEON UNITED STATES MARINE HOSPITAL SERVICE.

The Supervising Surgeon-General, Marine Hospital Service, Washington, D. C., under date of August 25, 1898, has issued the following circular:

A board of officers will be convened at Washington, Wednesday, November 9, 1898, for the purpose of examing candidates for admission to the grade of Assistant Surgeon in the United States Marine Hospital Service. It is desired that applications for this examination be made before November 1st.

Candidates must be between twenty-one and thirty years of age, graduates of a respectable medical college, and must furnish testimonials from responsible persons as to their character.

The following is the usual order of the examination: 1, Physical. 2, Written. 3, Oral. 4, Clinical.

In addition to the physical examination, candidates are required to certify that they believe themselves free from any ailment which would disqualify for service in any climate.

The examinations are chiefly in writing, and begin with a short autobiography by the candidate. The remainder of the written exercise consists in examination on the various branches of medicine, surgery, and hygiene.

The oral examination includes subjects of preliminary education, history, literature, and natural sciences.

The clinical examination is conducted at a hospital, and when practicable, candidates are required to perform surgical operations on the cadaver.

Successful candidates will be numbered according to their attainments on examination, and will be commissioned in the same order as vacancies occur.

Upon appointment, the young officers are, as a rule, first assigned to duty at one of the large marine hospitals, as at Boston, New York, New Orleans, Chicago, or San Francisco.

After five years' service, Assistant Surgeons are entitled to examinations for promotions to the grade of Passed Assistant Surgeon.

Promotion to the grade of Surgeon is made according to seniority, and after due examination, as vacancies occur in that grade. Assistant Surgeons receive sixteen hundred dollars; Passed Assistant Surgeons, two thousand dollars; and Surgeons, twenty-five hundred dollars per year. When quarters are not provided, commutation at the rate of thirty, forty, or fifty dollars a month, according to grade, is allowed.

